# Hospital-based management of predominant negative symptoms in schizophrenia: an observational study

**DOI:** 10.1017/S109285292610090X

**Published:** 2026-03-26

**Authors:** Jozef Dragašek, Zsofia Borbala Dombi, Péter László Herman, Viktor Dzurilla, Agota Barabassy

**Affiliations:** 1 1st Department of Psychiatry, Pavol Jozef Safarik University, Faculty of Medicine and University Hospital of Louis Pasteur, Košice, Slovakia; 2 Global Medical Division, Gedeon Richter Plc., Budapest, Hungary; 3 Gedeon Richter Slovakia, Bratislava, Slovakia

**Keywords:** Negative symptoms, schizophrenia, antipsychotic medication, observational study, inpatients

## Abstract

**Objective:**

The present study investigated the management of predominant negative symptoms in hospitalized patients and assess the impact of targeted pharmacological and psychological interventions.

**Methods:**

This longitudinal, prospective, multicenter cohort study was conducted across multiple hospitals in Slovakia, focusing on inpatients, with assessments at admission and discharge. Eligible participants were hospitalized adults (18–65) diagnosed with schizophrenia and predominant negative symptoms. Treatment effectiveness was measured using the modified Short Assessment of Negative Domain, Self-evaluation of Negative Symptoms, Personal and Social Performance, and Clinical Global Impression scales. Means changes and effect sizes were calculated from baseline to final assessment. Differences in the perception of negative symptoms between patients and doctors were examined.

**Results:**

At discharge, patients showed significant improvements in symptom severity and functioning on all scales. Primary and secondary negative symptoms significantly decreased, especially those linked to positive and affective symptoms. Functioning improved, with fewer severe impairments in daily life. Most patients were on antipsychotic polytherapy throughout hospitalization, with around 85% receiving multiple antipsychotics at admission and at discharge. Non-pharmacological interventions were also widely used, with nearly nine out of ten patients receiving at least one such therapy during hospitalization.

**Conclusions:**

Negative symptoms in schizophrenia pose a major treatment challenge, leading to functional impairment and poor quality of life. While positive symptoms often trigger hospitalizations, negative symptoms have a lasting impact on prognosis. Results suggest both D3-receptor–targeted pharmacotherapy, particularly cariprazine, and integrated non-pharmacological interventions may contribute to meaningful improvements in negative symptoms during hospital care.

## Introduction

Schizophrenia is a chronic, complex, and debilitating psychiatric disorder affecting approximately 1% of the global population.^1^ It has a profound impact on the daily functioning and quality of life of patients.^1^ The symptomatology of schizophrenia is multifaceted, encompassing five core symptom domains: positive symptoms such as hallucinations and delusions, negative symptoms like asociality or apathy, cognitive symptoms including disorganized thinking, affective symptoms such as depression and anxiety, and symptoms of hostility.^2^

### The importance of negative symptoms

Negative symptoms are among the most challenging aspects of schizophrenia, affecting approximately 60% of individuals diagnosed with the disorder.^3^ These symptoms are broadly categorized into two domains: expressive deficits, such as blunted affect and alogia, and experiential deficits, including avolition, anhedonia, and asociality.^4^^,^^5^ Collectively, these symptoms are also referred to as the “5As.”^6^ Negative symptoms can be either primary, meaning intrinsic to the disorder, or secondary, arising from positive or depressive symptoms or as a side effect of medication.^4^^,^^7^ Consequently, secondary negative symptoms may improve with treatment of their underlying causes, whereas primary negative symptoms are more persistent and associated with poorer long-term outcomes, including lower treatment adherence and diminished response to pharmacotherapy.^4^^,^^7^^,^^8^

The underlying neurobiology of negative symptoms is distinct from that of positive symptoms, with evidence suggesting that a hypodopaminergic state in the prefrontal cortex contributes to motivational deficits and diminished reward processing.^9^^,^^10^ Additionally, dysfunction in glutamatergic and serotonergic neurotransmission has been implicated in the pathophysiology of negative symptoms.^11^^,^^12^ Given their insidious onset and persistence, negative symptoms significantly impede patients’ ability to engage in social, vocational, and independent living activities.^3^

Historically, research and clinical efforts have prioritized positive symptoms due to their overt and distressing nature.^13^ However, emerging evidence underscores the crucial role of negative symptoms in determining long-term disability and social reintegration.^3^^,^^4^ The modern concept of recovery from schizophrenia emphasizes the importance of improving well-being, quality of life, and social functioning, all of which are adversely affected by negative symptoms.^8^ Furthermore, given their enduring nature, negative symptoms often persist even during periods of clinical stability and are frequently resistant to treatment.^14^

### The management of negative symptoms in hospital setting

Risk of hospitalization is associated with both positive and negative symptoms, as well as symptoms of hostility and aggression.^15^ While positive symptoms are often the primary focus of clinical intervention (as they contribute to acute psychiatric crises^16^), negative symptoms are linked to prolonged hospital stays and an increased risk of readmission.^17^ Indeed, a retrospective study by Vita and colleagues found that patients with more severe or predominant negative symptoms at discharge required more frequent and prolonged hospitalizations, as well as increased rehabilitative residential admissions during the 1-year follow-up.^18^ The study concluded that higher severity of negative symptoms was linked to greater psychiatric resource utilization, predicting increased long-term care needs compared to patients without predominant positive symptoms.^18^ Furthermore, another study of 429 subjects found that patients with severe negative symptoms used more high-cost psychiatric resources, including frequent hospital admissions and intensive residential care.^19^ Both primary and secondary negative symptoms contributed to greater psychiatric service use, with secondary symptoms particularly associated with higher-cost care.^19^ Consequently, the social and economic costs of schizophrenia are disproportionately high, with negative symptoms contributing to increased healthcare costs and resource utilization.^20^^,^^21^

Therefore, the management of negative symptoms in hospital settings presents a complex challenge.^22^^,^^23^ Despite their significant impact, these symptoms often receive less attention in inpatient psychiatric care, where the priority is typically stabilizing acute psychotic episodes and managing aggression risk.^24^ Effective management in the hospital setting requires a multidisciplinary approach that integrates pharmacological, psychological, and rehabilitative interventions.^25^ In addition, developing comprehensive treatment plans is essential to mitigating the impact of negative symptoms on patients’ recovery and long-term prognosis.^25^ There are no available guidelines for the acute treatment of negative symptoms in hospital settings.

### Pharmacological treatment of negative symptoms

Pharmacological treatments for negative symptoms are somewhat limited, with most antipsychotic medications providing only small to moderate reductions in these symptoms compared to placebo.^8^^,^^26^ Traditional antipsychotics have demonstrated minimal efficacy in alleviating negative symptoms, with some agents potentially exacerbating them due to dopaminergic blockade.^27^ Second- and third-generation antipsychotics, particularly those with partial dopamine D_3_-D_2_ agonist properties, such as cariprazine, aripiprazole, and risperidone,^28^^–^^32^ have shown greater promise in addressing negative symptoms.^8^ Among these newer antipsychotics, cariprazine features the highest affinity for D3 receptors.^33^^,^^34^ Indeed, Cerveri et al. recently proposed cariprazine as a first-line treatment due to its partial agonist effect on dopamine D_3_-D_2_ receptors.^35^ Beyond antipsychotic medications, other pharmacological strategies include the use of antidepressants and glutamatergic modulators.^26^ However, further research is needed to clarify the comparative effectiveness of different pharmacological agents and their long-term impact on functional recovery.

### Psychological treatment of negative symptoms

Psychosocial interventions play a crucial role in addressing negative symptoms. Approaches such as cognitive-behavioral therapy (CBT), social skills training, and vocational rehabilitation have demonstrated efficacy in enhancing motivation, social engagement, and overall functional outcomes.^36^^,^^37^ CBT tailored for negative symptoms specifically targets motivational deficits and fosters engagement in meaningful activities.^38^ Additionally, structured rehabilitation programs incorporating occupational therapy and supported employment initiatives are essential in promoting functional recovery.^39^^,^^40^ Integrating these interventions with pharmacotherapy may provide a more comprehensive treatment approach, addressing the multifaceted nature of negative symptoms and optimizing long-term outcomes.^41^

### Aims

Given the substantial impact of negative symptoms on patients’ functioning and quality of life, a nuanced understanding of these symptoms and their management is essential for developing more effective treatment strategies. Therefore, the primary aim of this study was to examine the therapeutic interventions employed for the treatment of predominant negative symptoms in hospital settings and to deepen our understanding of the relationship between negative symptoms and psychosocial functioning.

## Methods

### Study design

By design, the present study was a longitudinal, prospective, multicentric cohort study involving 14 hospitals in Slovakia. The study was divided into two arms, an outpatient, and an inpatient arm. The results of the outpatient arm were published by the same authors in 2024.^42^ The present article focuses on the inpatient arm of the study, for which data was collected during hospitalization, with assessments performed at two time points: admission and discharge. The cohort study received approval by the Ethics Committee of the Košice Self-Governing Region (3618/2020/ODDZ-07169) and informed written consent was obtained from all participants. The study complies with the Declaration of Helsinki. Safety parameters and adverse events were monitored and managed as in standard clinical practice.

### Patient characteristics

Hospitalized patients between the age of 18–65, who fulfilled the criteria for schizophrenia diagnosis as outlined by the International Classification of Diseases 10th edition^43^ were suitable to participate in the study. Additionally, patients also had to have predominant negative symptoms in order to be included. The European Psychiatric Association’s (EPA) guidance for negative symptoms was utilized to assess predominant negative symptoms, which requires the presence of at least two symptoms out of the five “As”: anhedonia, avolition, alogia, affective blunting, and asociality.^4^^,^^8^ The presence of predominant negative symptoms was decided by the physicians in the hospital at admission based on the patients’ anamnesis.

### Outcome measures

#### Symptom composition

Primary and secondary negative symptoms were evaluated by clinicians and differentiated using a structured interview based on the guidance provided by the EPA.^4^ Symptom composition with a special focus on negative symptoms were assessed by the following measures: modified Short Assessment of Negative Domain (m-SAND) scale, the Self-evaluation of Negative Symptoms (SNS) scale^44^ (focusing on negative symptomatology) and the Clinical Global Impression Severity (CGI-S) and Improvement (CGI-I) scales^45^ (focusing on overall psychopathology). The original SAND scale was used in a Latvian study on cariprazine’s effectiveness in patients with predominant negative symptoms.^46^ This anamnesis-based scale includes seven items: two positive (SAND-P) and five negative (SAND-N) items, rated from 0 to 6.^46^ Due to difficulty distinguishing ‘minimal’ from “mild,” a modified m-SAND was decided to be used with changing the scoring to 1–6 while retaining the same items.^42^ Besides the m-SAND, the SNS, a self-administered questionnaire that measures five domains of negative symptoms (the 5As) from the patient’s perspective was also administered. The Slovak-language SNS was culturally and linguistically adapted using a forward–backward translation by two independent experts, back-translation, independent review, and final reconciliation approved by the instrument’s author. The adapted version has been disseminated for routine clinical use in Slovakia. A formal psychometric validation on a Slovak cohort has not yet been published. Finally, to evaluate overall disorder severity and improvement throughout the hospital stay the CGI-S and CGI-I was used. In the study, CGI-I was captured with a study-specific orientation (higher score = improvement), which was later harmonized with the original scoring by reversing the items.

#### Functioning

Psychosocial functioning was assessed primarily via the Personal and Social Performance (PSP) scale.^47^ The PSP scale evaluates social functioning in psychiatric disorders, independent of symptoms by focusing on four areas: socially useful activities, relationships, self-care, and aggressive behaviours.^47^ Besides the PSP, patient characteristics such as employment status, disability status, and disorder insight were also documented. Disorder insight was evaluated by the clinician at baseline and discharge based on available sources: the patient interview, informant or caregiver report, and routine clinical documentation.

#### Treatment

For each patient, the treatment patterns at admission and discharge were noted for both antipsychotic treatment and psychotherapies, alongside with information on adherence and treatment duration. Adherence was recorded based on available sources: the patient interview, informant or caregiver report, and routine clinical documentation.

### Statistical analyses

Patient and treatment characteristics were summarized with descriptive statistics (means, percentages, and standard deviations). To understand the changes in symptom composition between hospital admission and discharge, mean changes were calculated for the following measures: m-SND, SNS, PSP, and CGI-S using a mixed model for repeated measures. A Bland–Altman plot was generated to compare patients’ and physicians’ perspectives on negative symptoms by examining the agreement between SNS scores (patient-rated) and m-SAND negative sub-scores (clinician-rated). All analyses were conducted using Statistical Analysis Software.

## Results

### Admission

A total of 175 patients were eligible to be included in the study who were admitted to hospital with a diagnosis of schizophrenia and exhibited predominant negative symptoms according to clinical judgement.

#### Patient characteristics

The mean age of patients was 39.9 years and 64% of them were male (Table 1). Most patients were unemployed (74.9%), and only a minority of patients worked occasionally (7.4%) or had a part-time job (6.9%). In terms of disability status, more than two third of the patients were disabled primarily due to their psychiatric condition (70.3%). On average, patients were ill for more than 13 years (Table 2). Most of them had a diagnosis of paranoid schizophrenia (63.4%), residual schizophrenia (11.4%), or undifferentiated schizophrenia (11.4%). About one third of the patients also had psychiatric comorbidity (37.7%); 22.9% had comorbid substance abuse, 9.7% had comorbid depression, and 7.4% had comorbid personality disorder. Somatic comorbidities were also present (34.3%), especially hypertension (16.0%) and obesity (6.3%). In terms of disorder insight, the majority of patients had partial (46.9%) or no insight at all (42.9%).

**Table 1.** Demographic Characteristics

Population	
Intention to treat population, n (%)	175 (100)
Demographics	
Age, mean (SD), y	39.9 (12.4)
Males, n (%)	112 (64.0)
Employment status	
Unemployed	131 (74.9)
Occasionally working	13 (7.4)
Part time job	12 (6.9)
Full-time job	8 (4.6)
Pensioner	8 (4.6)
Student	3 (1.7)
Disability status	
Yes, primary from psychiatric indication	123 (70.3)
No	51 (29.1)
Yes, primary from non-psychiatric indication	1 (0.6)

**Table 2.** Disorder Characteristics

Schizophrenia characteristics	
Duration of illness, mean (SD), y	13.7 (10.1)
Schizophrenia diagnosis, n (%)^a^	
Paranoid schizophrenia	111 (63.4)
Residual schizophrenia	20 (11.4)
Undifferentiated schizophrenia	20 (11.4)
Comorbidities	
Psychiatric comorbidity, n (%)^a^	66 (37.7)
Substance abuse	40 (22.9)
Depression	17 (9.7)
Personality disorder	13 (7.4)
Somatic comorbidity, n (%)^a^	60 (34.3)
Hypertension	28 (16.0)
Obesity	11 (6.3)
Insight, n (%)	
Full insight	18 (10.3)
Partial insight	82 (46.9)
No insight	75 (42.9)

a Only above 5% displayed.

#### Symptom composition

As per protocol, all patients exhibited predominant negative symptoms (100%), however, in only 58.9% of the patients was the presence and severity of negative symptoms the reason for hospitalization (Table 3). At admission, almost all patients had blunted affect (96.6%), and many of them also presented with avolition (89.1%), asociality (84%), anhedonia (83.4%), and alogia (61.1%) according to clinical judgement. Secondary negative symptoms were also prevalent (42.9%) mostly due to affective (36.7%) and positive symptoms (28.6%) as well as due to adverse drug reactions (21.2%).

**Table 3.** Negative Symptom Characteristics at Admission and Discharge

Predominant negative symptoms, n (%)		
Presence	175 (100.0)	
Reason for hospitalisation, n (%)	103 (58.9)	
Primary negative symptoms, n (%)	Presence at admission	Improvement at discharge
Affective blunting	169 (96.6)	143 (84.6)
Alogia	107 (61.1)	78 (72.9)
Avolition, apathy	156 (89.1)	137 (87.8)
Anhedonia	146 (83.4)	127 (87.0)
Asociality	147 (84.0)	118 (80.3)
Secondary negative symptoms, n (%)		
Total	75 (42.9)	67 (89.3)
Due to positive symptoms	50 (28.6)	49 (65.3)
Due to affective symptoms	45 (36.7)	44 (58.7)
Due to adverse drug reactions	24 (21.2)	25 (33.3)

The mean m-SAND total score was 27.1, reflecting a severe symptom profile (Table 4). While positive symptoms were generally mild to moderate in severity (mean m-SAND Positive score = 5.7), negative symptom sub-scores ranged from severe to very severe (mean m-SAND Negative item scores = 4.3–4.7), with the exception of alogia, which was rated as moderate (mean score = 3.5). Notably, patients perceived avolition and alogia as the most severe symptoms (mean scores = 5.3 and 5.0, respectively), followed by asociality (mean: 4.8), affective blunting (mean = 4.6), and anhedonia (mean = 4.3), as indicated by the mean SNS scores. Clinicians’ overall assessment reinforced this, with a mean CGI-S score of 5.1, indicating marked illness.

**Table 4.** Treatment Effectiveness

	ADMISSION mean (SD)	DISCHARGE mean (SD)	Mean change (SD)	Effect size (ES)	95% CI	*p*-value
m-SAND total	27.13 (5.33)	15.91 (5.65)	−11.22 (5.79)	−2.74 (0.149)	(−3.03, −2.44)	*< 0.001*
m-SAND-P	5.68 (2.79)	3.19 (1.87)	−2.49 (2.24)	−1.57 (0.122)	(−1.81, −1.33)	*< 0.001*
Hallucinations	2.39 (1.53)	1.38 (0.93)	−1.01 (1.26)	−1.13 (0.115)	(−1.36, −0.9)	*< 0.001*
Delusions	3.29 (1.61)	1.81 (1.12)	−1.47 (1.33)	−1.57 (0.122)	(−1.81, −1.33)	*< 0.001*
m-SAND-N	21.45 (4.14)	12.72 (4.40)	−8.73 (4.79)	−2.58 (0.145)	(−2.86, −2.29)	*< 0.001*
Anhedonia	4.29 (1.13)	2.60 (1.03)	−1.69 (1.27)	−1.88 (0.129)	(−2.13, −1.63)	*< 0.001*
Affective blunting	4.73 (0.89)	2.92 (0.91)	−1.81 (1.15)	−2.21 (0.136)	(−2.48, −1.95)	*< 0.001*
Avolition, apathy	4.62 (1.04)	2.57 (1.01)	−2.05 (1.25)	−2.32 (0.139)	(−2.59, −2.05)	*< 0.001*
Alogia	3.47 (1.56)	2.19 (1.18)	−1.28 (1.35)	−1.34 (0.118)	(−1.57, −1.1)	*< 0.001*
Asociality	4.34 (1.37)	2.43 (1.16)	−1.91 (1.42)	−1.91 (0.129)	(−2.16, −1.65)	*< 0.001*
PSP total						
functioning is poor (30–0)	112 (64%)	10 (6%)	–	–	–	–
manifest disabilities (70–31)	60 (34%)	123 (70%)	–	–	–	–
only mild difficulties (100–71)	3 (2%)	42 (24%)	–	–	–	–
PSP items						
Socially useful activities	4.61 (1.11)	3.02 (1.08)	−1.58 (1.31)	−1.71 (0.125)	(−1.96, −1.47)	*< 0.001*
Personal and social relationships	4.61 (0.98)	2.85 (1.00)	−1.77 (1.20)	−2.09 (0.133)	(−2.35, −1.82)	*< 0.001*
Self-care	3.94 (1.20)	2.09 (1.03)	−1.85 (1.36)	−1.92 (0.129)	(−2.17, −1.66)	*< 0.001*
Disturbing and aggressive behaviour	2.80 (1.67)	1.29 (0.69)	−1.51 (1.60)	−1.34 (0.118)	(−1.57, −1.11)	*< 0.001*
SNS total	23.98 (8.20)	16.17 (7.95)	−8.33 (7.47)	−1.52 (0.141)	(−1.79, −1.24)	*< 0.001*
Asociality/items 1–4	4.78 (2.48)	2.99 (1.97)	−1.98 (2.09)	−1.3 (0.137)	(−1.57, −1.03)	*< 0.001*
Affective blunting/items 5–8	4.62 (1.73)	3.44 (1.65)	−1.15 (1.64)	−0.99 (0.132)	(−1.25, −0.73)	*< 0.001*
Alogia/items 9–12	5.02 (1.97)	3.37 (1.82)	−1.74 (1.99)	−1.19 (0.134)	(−1.46, −0.93)	*< 0.001*
Avolition/items 13–16	5.26 (2.11)	3.41 (1.98)	−1.93 (1.96)	−1.37 (0.138)	(−1.64, −1.1)	*< 0.001*
Anhedonia/items 17–20	4.32 (2.14)	2.95 (2.05)	−1.53 (1.75)	−1.18 (0.136)	(−1.45, −0.91)	*< 0.001*
CGI-I	–	2.51 (0.90)	–	–	–	–
CGI-S	5.13 (1.04)	3.38 (1.36)	−1.75 (1.41)	−1.76 (0.126)	(−2.01, −1.51)	*< 0.001*

Abbreviations: CGI-I, clinical global impressions-improvement; CGI-S, clinical global impressions-severity; ES, effect size; LS, least squares; PSP, the personal and social performance scale; m-SAND, modified short assessment of negative domains; m-SAND-N, modified short assessment of negative domains negative symptom sub-scale; m-SAND-P, modified short assessment of negative domains positive symptom sub-scale; SNS, self-evaluation of negative symptoms; SD, standard deviation; SE, standard error.

Statistical significance was set at *p* < 0.05.

**Figure 1.**
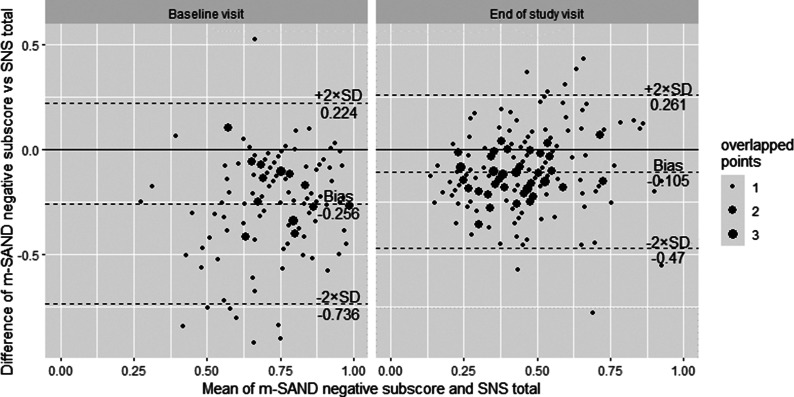
Bland–Altman agreement plot: difference between SAND Negative Sub-Score and SNS Total score (or change) versus their average scores are expressed as % of the corresponding max value.

When comparing the view of patients on negative symptoms compared to their physicians, the Bland–Altman plot (Figure 1) revealed a negative bias of −0.256, indicating that clinicians consistently rated negative symptoms lower than patients did. The limits of agreement ranged from −0.736 to 0.224, reflecting moderate variability in the level of agreement between the two rating sources.

#### Functioning

The majority of patients exhibited poor functioning (64.0%) based on the PSP total score, with one-third classified under the ‘manifest disabilities’ category (Table 4). Only three patients were considered to have mild difficulties. Among the individual domains, the most pronounced impairments were observed in socially useful activities and personal and social relationships, both with a mean score of 4.6. Self-care also seemed to pose a significant challenge, with a mean score of 3.9.

### Discharge

At discharge, patients showed substantial reduction in both positive and negative symptoms as well as marked improvement in functioning as indicated by the shifts in PSP total scores (Table 5).

**Table 5.** Treatment Characteristics

Treatment duration, mean (SD) in days				
Duration of hospital stay	37.75 (23.9)			
Duration of pharmacotherapy	29.85 (19.9)			
Adherence				
Full	31 (17.7)			
Partial	83 (47.4)			
Insufficient	61(34.9)			
Pharmacotherapy	ADMISSION		DISCHARGE	
Antipsychotic monotherapy	25 (14.3)		27 (15.4)	
Antipsychotic polytherapy	150 (85.7)		148 (84.6)	
Non-pharmacological treatment	159 (90.9)		151 (86.3)	
Work therapy	103 (58.9)		119 (68.0)	
Practicing a social skills	94 (53.7)		112 (64.0)	
other types of psychotherapy	65 (37.1)		73 (41.7)	
Supportive psychotherapy	59 (33.7)		69 (39.4)	
Electroconvulsive therapy	32 (18.3)		27 (15.4)	
Other	35 (20.0)		27 (15.4)	
Type of antipsychotic, n (%)	Mono	Poly	Mono	Poly
Cariprazine	12 (6.9)	141 (80.6)	20 (11.4)	146 (83.4)
Olanzapine	4 (2.3)	47 (26.9)	3 (1.7)	46 (26.3)
Haloperidol	1 (0.6)	39 (22.3)	0 (0.0)	15 (8.6)
Clozapine	6 (3.4)	27 (15.4)	1 (0.6)	44 (25.1)
Quetiapine	0 (0.0)	21 (12.0)	0 (0.0)	17 (9.7)
Paliperidone palmitate	0 (0.0)	13 (7.4)	1 (0.6)	10 (5.7)
Flupentixol	0 (0.0)	11 (6.3)	0 (0.0)	11 (6.3)
Levomepromazine	0 (0.0)	8 (4.6)	0 (0.0)	5 (2.9)
Risperidone	0 (0.0)	8 (4.6)	0 (0.0)	3 (1.7)
Aripiprazole	0 (0.0)	7 (4.0)	0 (0.0)	6 (3.4)
Paliperidone	1 (0.6)	7 (4.0)	1 (0.6)	7 (4.0)
Chlorprothixene	0 (0.0)	5 (2.9)	0 (0.0)	2 (1.1)
Zuclopenthixol	1 (0.6)	4 (2.3)	1 (0.6)	4 (2.3)
Zipresidone	0 (0.0)	2 (1.1)	0 (0.0)	2 (1.1)
Ziprasidone	0 (0.0)	2 (1.1)	0 (0.0)	0 (0.0)
Fluphenazine	0 (0.0)	1 (0.6)	0 (0.0)	2 (1.1)
Amisulpride	0 (0.0)	1 (0.6)	0 (0.0)	2 (1.1)
Dual combinations, n (%)^a^				
Cariprazine + Olanzapine	34 (19.4)		41 (23.4)	
Cariprazine + Clozapine	16 (9.1)		37 (21.1)	
Cariprazine + Haloperidol	13 (7.4)		5 (2.9)	
Cariprazine + Paliperidone palmitate	11 (6.3)		10 (5.7)	
Cariprazine + Quetiapine	10 (5.7)		11 (6.3)	
Cariprazine + Risperidone	5 (2.9)		3 (1.7)	
Cariprazine + Paliperidone	4 (2.3)		4 (2.3)	
Trial combinations, n (%)^a^				
Cariprazine + Haloperidol + Olanzapine	5 (2.9)		2 (1.1)	
Cariprazine + Haloperidol + Clozapine	5 (2.9)		0 (0.0)	
Cariprazine + Aripiprazole + Quetiapine	4 (2.3)		2 (1.1)	

a Taken by more than 2% of patients.

#### Symptom composition

Improvement rates at discharge were high across primary negative symptoms, with 84.6% of patients improving in affective blunting, 72.9% in alogia, 87.8% in avolition/apathy, 87.0% in anhedonia, and 80.3% in asociality (Table 3). Additionally, secondary negative symptoms improved in 89.3% of cases, including those due to positive symptoms (65.3%) and affective symptoms (58.7%), though those related to adverse drug reactions remained relatively stable (33.3%).

**Figure 2.**
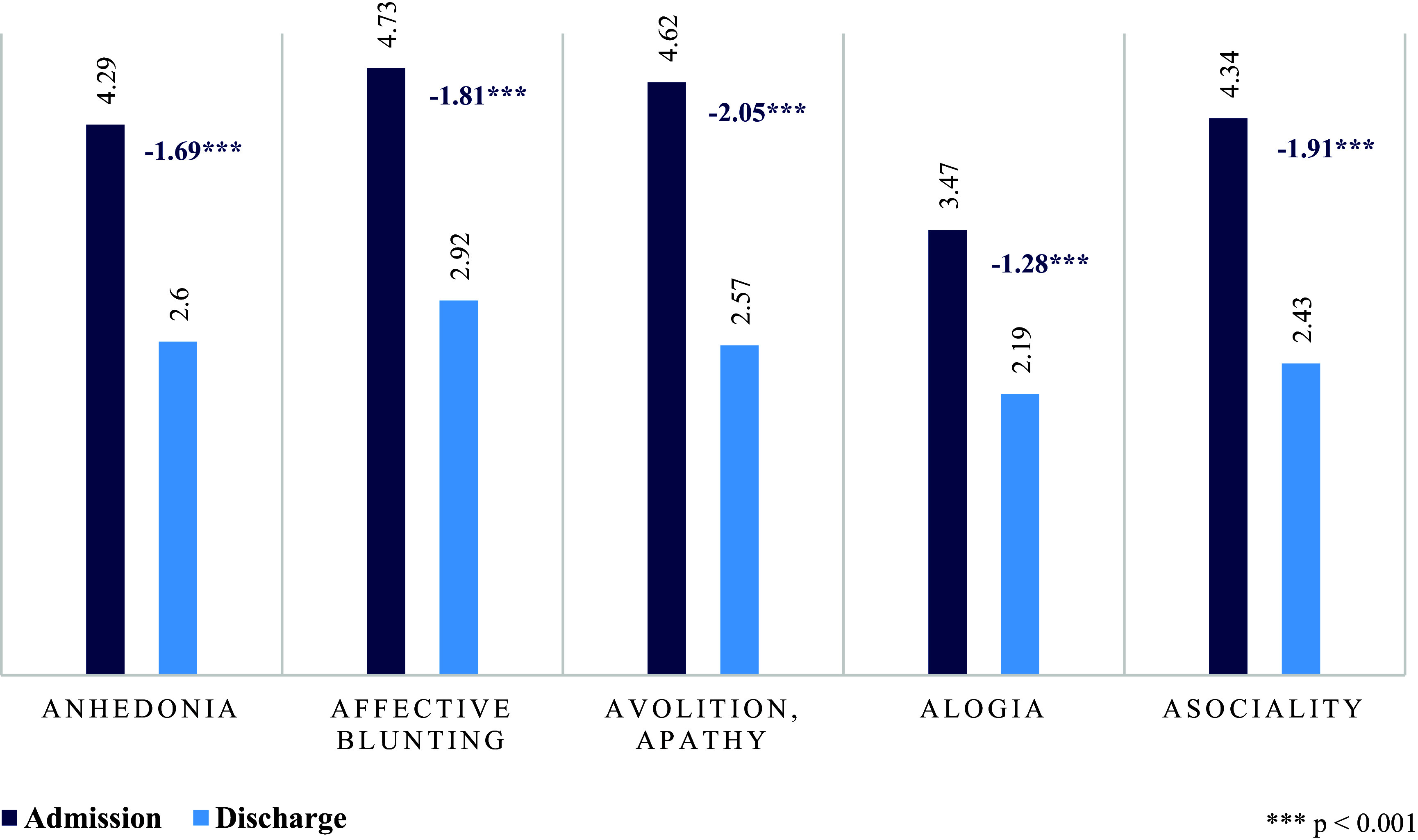
Mean change from admission to discharge in m-SAND Negative sub-scale item scores.

**Figure 3.**
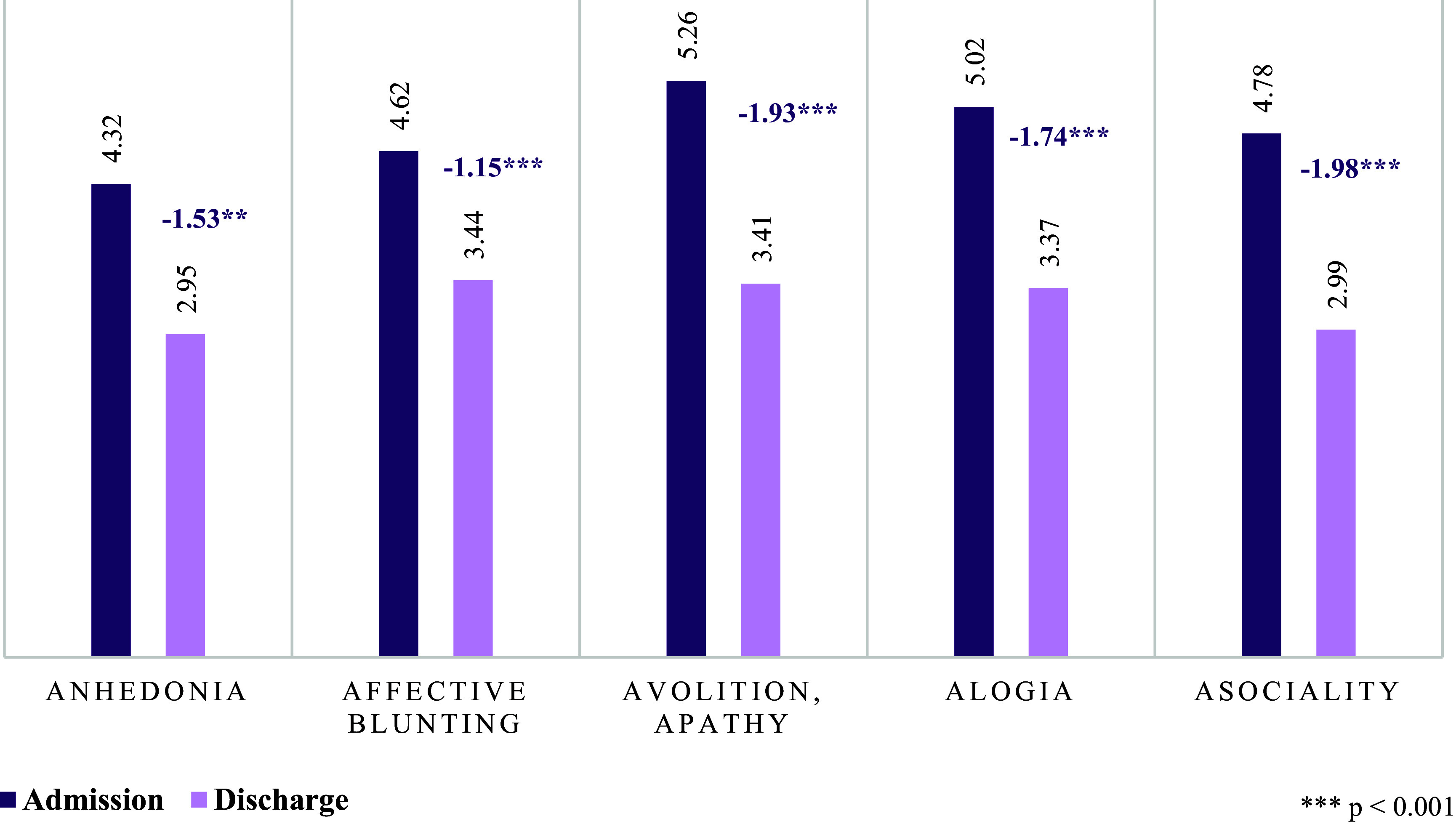
Mean change from admission to discharge in SNS item scores.

The m-SAND total score decreased significantly from 27.13 to 15.91 (mean change = −11.22, ES = −2.74, *p < 0.001*), with reductions in both positive symptoms (m-SAND-P: -2.49, ES = −1.57, *p < 0.001*) and negative symptoms (m-SAND-N: -8.73, ES = −2.58, p < 0.001) (Table 4). Among positive symptoms, both hallucinations and delusions improved significantly (mean change = −1.01 and − 1.47, respectively, *p < 0.001*). Within negative symptoms, the greatest reductions were seen in avolition (mean change = −2.05, *p < 0.001*), asociality (mean change = −1.91, *p < 0.001*), and affective blunting (mean change = −1.81, *p < 0.001*) (Figure 2). The SNS total score also improved (mean change = −8.33, ES = −-1.52, *p < 0.001*), confirming these improvements across multiple assessment scales. Patients indicated the biggest improvement in asociality (mean change = −1.98, *p < 0.001*), avolition (mean change = −1.93, *p < 0.001*), and alogia (mean change = −1.74*, p < 0.001*) (Figure 3).

At discharge, the Bland–Altman plot demonstrated a reduced mean difference (bias) of −0.105 between clinician-rated m-SAND negative sub-scores and patient-rated SNS total scores, indicating a smaller discrepancy in symptom perception compared to baseline (Figure 1). The limits of agreement ranged from −0.470 to +0.261, reflecting slightly improved consistency the two measures. The majority of data points clustered around the mean, with fewer extreme differences, suggesting enhanced agreement. A leftward shift along the x-axis was observed, indicating that both patients and clinicians reported a reduction in the severity of negative symptoms over the course of treatment. Additionally, a slight upward shift in the differences suggests a reduced tendency among clinicians to underscore symptom severity relative to patients, highlighting a convergence in symptom perception by the end of the study.

#### Functioning

At admission, 64.0% of patients had poor functioning, while this was reduced to only 6.0% at discharge (Table 4). The proportion of patients with manifest disabilities (PSP 70–31 range) increased from 34.3 to 70.0%, and those with only mild difficulties (PSP 100–71 range) rose from 1.7 to 24.0%. Improvements were particularly notable in self-care (mean change = −1.85, ES = −1.92, *p > 0.001*), and personal and social relationships (−1.77, ES = −2.09, *p < 0.001*). The CGI-S score decreased from 5.13 to 3.38 (mean change = −1.75, ES = −1.76, *p < 0.001*), while the CGI-I score at discharge was 2.51, reflecting substantial clinical improvement.

### Treatment

The average duration of hospitalization was 37.8 days (SD = 23.9), while patients received pharmacotherapy for an average of 29.9 days (SD = 19.9) (Table 5). Adherence to treatment varied, with 17.7% of patients demonstrating full adherence, nearly half (47.4%) showing partial adherence, and 34.9% classified as having insufficient adherence.

#### Pharmacotherapy

The majority of patients were on antipsychotic polytherapy throughout hospitalization, with 85.7% receiving multiple antipsychotics at admission and 84.6% at discharge (Table 5). Monotherapy was less common but showed a slight increase from 14.3% at admission to 15.4% at discharge.

**Figure 4.**
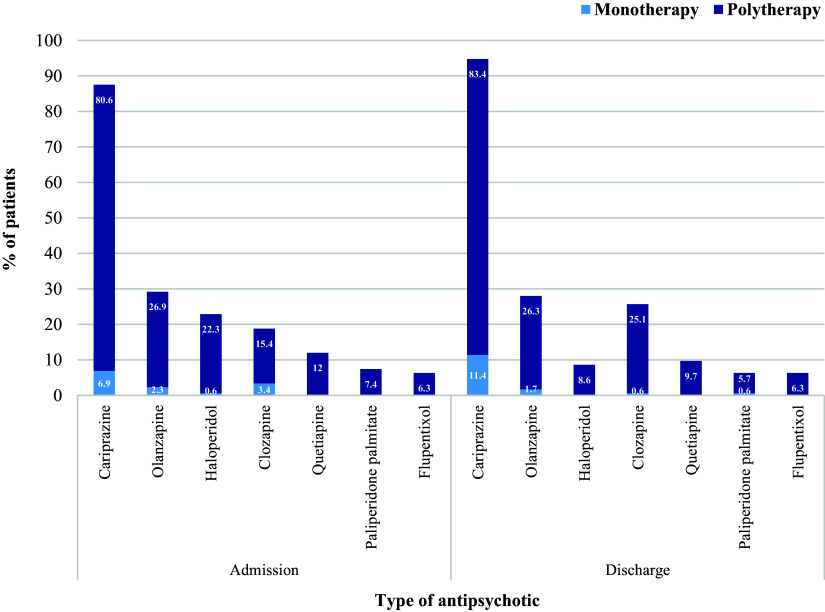
Monotherapy and polytherapy patterns at admission and discharge.

To address the overwhelming presence of negative symptoms, cariprazine was the most frequently prescribed antipsychotic, particularly in combination therapy (80.6% at admission, 83.4% at discharge), with an increasing trend in monotherapy use (6.9 to 11.4%) (Figure 4). Clozapine use in monotherapy decreased (3.4 to 0.6%), whereas its use in polytherapy increased (15.4 to 25.1%), reflecting a potential strategy for managing treatment-resistant symptoms. Haloperidol saw a significant reduction in polytherapy use from 22.3 to 8.6%, possibly due to switching strategies. Other antipsychotics, such as olanzapine, quetiapine, paliperidone palmitate, and risperidone, maintained relatively stable usage rates. Some agents, including ziprasidone and chlorprothixene, were used minimally, with little to no change over time.

In terms of the specific combinations, the most frequently used dual antipsychotic combination was cariprazine + olanzapine, prescribed in 19.4% of cases at baseline and 23.4% at discharge, followed by cariprazine + clozapine (9.1% at baseline and 21.1% at discharge) and cariprazine + haloperidol (7.4% at baseline and 2.9% at discharge) (Table 5). Less commonly used combinations included cariprazine + paliperidone palmitate (6.3% at baseline and 5.7% at discharge) and cariprazine + quetiapine (5.7% at baseline and 6.3% at discharge). Triple combinations were relatively rare, each occurring in fewer than 3% of cases, with cariprazine + haloperidol + olanzapine and cariprazine + haloperidol + clozapine being the most common.

#### Non-pharmacological treatment

Non-pharmacological treatments were widely used, though slightly less frequent at discharge (90.9% at admission versus 86.3% at discharge) (Table 5). Work therapy and social skills training saw notable increases over the course of hospitalization (58.9 to 68.0% and 53.7 to 64.0%, respectively). Various psychotherapeutic approaches, including supportive psychotherapy, also became more prevalent at discharge. Electroconvulsive therapy was administered to 18.3% of patients but was reduced to 15.4% by discharge.

## Discussion

In summary, patients showed notable improvements in both symptom severity and overall functioning at discharge. Negative symptoms improved in most patients, with primary negative symptoms showing substantial reductions. Secondary negative symptoms, particularly those linked to positive and affective symptoms, also decreased. An overall improvement in the agreement of negative symptom assessment between patients and clinicians were detected at discharge, nonetheless clinicians still tended to slightly underestimate negative symptoms relative to patients’ self-reports. Functioning significantly improved, with fewer patients experiencing severe impairments in socially useful activities, personal and social relationships, and self-care. Treatment patterns remained stable, with most patients receiving antipsychotic polytherapy (with cariprazine being the most commonly prescribed agent) and non-pharmacological interventions. Adjustments in pharmacotherapy included increased monotherapy use for certain agents and reduced reliance on haloperidol in polytherapy, possibly reflecting efforts to optimize treatment efficacy and tolerability. The adherence to treatment varied, with some achieving full compliance and others showing partial or insufficient adherence.

### Comparison to the outpatient arm of the study

In comparing the two study arms, we found that the two groups were highly similar in terms of total patient number and demographic characteristics such as mean age and the percentage of male patients.^42^ Diagnostic trends were also comparable, with most patients having paranoid schizophrenia, followed by residual schizophrenia.^42^ Regarding comorbidities, both groups exhibited substance use disorder, depression, and personality disorders, though these were more prevalent in the hospital arm.^42^ Similarly, somatic comorbidities such as hypertension and obesity were present in both groups, with hypertension being more frequent among hospitalized patients.^42^ Employment status differed significantly, as unemployment was higher in the hospital arm.^42^ As expected, insight was lower in hospitalized patients, 42% had no insight compared to just 0.2% in the outpatient group.^42^

Symptom severity was consistently higher in the hospital arm. At baseline, hospitalized patients had higher total scores across all symptom domains, including functioning, where 64% had poor functioning according to the total PSP scores compared to 45% in the outpatient group.^42^ Interestingly, self-rated negative symptoms were initially higher in the outpatient group, but by discharge and at the one-year follow-up, the differences had narrowed.^42^ While the overall mean change in negative symptoms was slightly greater in the hospital arm, this was from a higher baseline.^42^ CGI scores also reflected more severe illness and greater improvement in the hospital arm.^42^

Higher self-rated negative symptoms in the outpatient group at baseline may be due to better insight and the ability to identify and describe subjective symptoms more accurately. Outpatients, who are generally more stable and better integrated, often have greater insight into their illness and can more thoroughly assess their negative symptoms, such as avolition, anhedonia, or asociality.^48^ In contrast, hospitalized patients may have reduced insight upon admission, which is typical in more severe cases of schizophrenia, especially when intense positive symptoms like hallucinations and delusions are present.^48^ These acute psychotic manifestations may impair patients’ ability to objectively assess their negative symptoms, leading to an underestimation at admission. As positive symptoms are managed during hospitalization and insight improves, hospitalized patients may become better able to reflect on their negative symptoms, potentially narrowing the gap between the groups at discharge and after 1 year of follow-up.

In terms of treatment, monotherapy was slightly more common in the outpatient arm than in the hospital arm.^42^ The antipsychotic treatment patterns were similar in both settings, with cariprazine being the most frequently prescribed, primarily in combination therapy.^42^ Non-pharmacological treatments also showed only slight differences: hospitalized patients primarily engaged in occupational therapy and social skills training, whereas outpatients most commonly received supportive psychotherapy, social skills training, and occupational therapy.^42^

Finally, outpatients demonstrated both a higher baseline level of insight and significant improvement over time, while inpatients had a higher proportion with no insight and much lower rates of full insight. These differences may reflect disparities in illness severity, treatment continuity, or the therapeutic context.

### Functioning and negative symptoms

This study reinforces the established link between negative symptoms and overall functioning in individuals with schizophrenia. Our findings indicate that as negative symptoms improved, there was a corresponding enhancement in patients’ functional abilities.^49^^,^^50^ Notably, the hospital setting, with an average inpatient stay of 37 days, facilitated significant reductions in negative symptoms when appropriate treatment strategies were employed. This duration aligns with previous research, which has reported mean hospital stays ranging from approximately 18 to 128 days for patients with schizophrenia.^51^^–^^53^ These results underscore the potential for meaningful clinical improvements within a relatively short inpatient period.

### The role of cariprazine in negative symptoms and functioning

According to the results, cariprazine, a dopamine D_3_-D_2_ receptor partial agonist, was utilized the most both as poly- and monotherapy. These findings are consistent with the treatment algorithm proposed by Cerveri et al.,^35^ which recommends cariprazine as a first-line medication for the treatment of negative symptoms. Additionally, the results further validate the claim that this algorithm has been implemented in Slovakia, as confirmed by a review involving 17 experts from the Central and Eastern European region.^54^

Indeed, cariprazine has previously been coined as a promising treatment option for negative symptoms in literature.^28^^,^^29^ Clinical trials have demonstrated that cariprazine significantly improves negative symptoms compared to placebo and other antipsychotics.^55^ Notably, results of a 26-week, randomized, double-blind, clinical trial demonstrated that cariprazine showed significant improvement in predominant negative symptoms of schizophrenia compared to risperidone in clinically stable patients, suggesting a distinct efficacy on this symptom domain.^28^ Additionally, a 16-week open-label observational study in Latvia corroborated these findings, where the effectiveness and safety of cariprazine in 116 schizophrenia patients with predominant negative symptoms and insufficient response to prior antipsychotics was evaluated. In this real-world study, cariprazine treatment led to significant improvements in negative symptoms and overall illness severity, with over 70% of patients showing clinical improvement, while adverse events were reported in 40% of patients.^46^ Case studies further illustrate its clinical application in managing negative symptoms, providing insights into its therapeutic role in both chronic and first-episode schizophrenia.^56^^–^^58^ Furthermore, real-world evidence suggests that cariprazine is associated with fewer metabolic adverse effects compared to other second-generation antipsychotics.^59^^,^^60^

Although primarily intended for monotherapy, polytherapeutic applications of cariprazine are increasingly described in the literature. In the present study, both dual and trial combinations were observed. The most common dual combinations included cariprazine with olanzapine and clozapine, followed by paliperidone palmitate, quetiapine, and, less frequently, haloperidol. In the latter case, the combination with haloperidol seem to represent a cross-titration strategy, where haloperidol was gradually tapered while cariprazine was continued as monotherapy. Trial combinations, such as cariprazine–haloperidol–olanzapine or cariprazine–haloperidol–clozapine, were also noted, but in most cases, these also appeared to reflect temporary titration regimens, eventually consolidating into dual or single-agent therapy.

From a pharmacological perspective, these patterns can be rationalized in light of the complementary receptor binding profiles of cariprazine and other antipsychotics. A recent guideline outlined the rationale for antipsychotic polypharmacy based on receptor profile complementarity, highlighting that third-generation antipsychotics such as cariprazine may be particularly suited for this purpose.^61^ Cariprazine is a dopamine partial agonist, but with a distinctive pharmacological profile characterized by a higher D_3_ affinity and longer half-life.^61^ This pharmacodynamic profile suggests that adjunctive use with agents such as clozapine, olanzapine, or quetiapine—antipsychotics with weaker D_3_ engagement—may result in clinically advantageous complementarity.^61^ Indeed, available case reports suggest beneficial effects of cariprazine–clozapine combinations in treatment-resistant schizophrenia and patients with persistent negative symptoms.^57^^,^^62^^–^^64^

Taken together, both our observational data and the emerging literature indicate that cariprazine may represent a promising option in selected cases of antipsychotic polypharmacy. While current evidence remains limited to case reports and naturalistic studies, the complementary pharmacological rationale and preliminary clinical experience suggest that combinations such as cariprazine with clozapine, olanzapine, or quetiapine may be particularly worthy of further exploration. Larger, high-quality randomized controlled trials will be needed to establish the clinical utility of these strategies.

### Limitations

Several limitations must be acknowledged in this observational study. The absence of a control group precludes definitive conclusions regarding treatment efficacy and patient outcomes, and the lack of information on prior antipsychotic treatment as well as concomitant medications (eg benzodiazepines, mood stabilizers, and antidepressants) further limits the interpretability of the findings. Additionally, the m-SAND scale, one of our primary outcome measures, lacks formal validation.^42^^,^^46^ However, it is derived from the well-established CGI-S scale, which is known for its good inter-rater reliability and widespread use in other studies.^42^^,^^46^ Finally, the observational design also introduces potential biases, including observer bias, inter-rater variability, information bias, and measurement bias, which may affect the internal validity of our findings.^65^^,^^66^

### Future research

Future studies should aim to identify the most effective treatment combinations in hospital settings to improve both functioning and negative symptoms in patients with schizophrenia. Randomized controlled trials with larger, more diverse populations are necessary to establish causality and generalize findings. Investigating the long-term effects of various treatment strategies on patient outcomes post-discharge would also provide valuable insights into sustained symptom management and functional recovery.

## Conclusion

Negative symptoms represent a critical treatment challenge in schizophrenia, contributing to significant functional impairment and poor quality of life.^3^ While positive symptoms often drive acute hospitalizations, negative symptoms have a more profound and enduring impact on patients’ long-term prognosis.^17^^–^^19^ Emerging evidence supports the use of novel pharmacological treatments, such as cariprazine, in addressing negative symptoms and improving functional outcomes.^28^^,^^46^^,^^67^ Additionally, integrated psychosocial interventions are essential in optimizing recovery and enhancing patients’ ability to reintegrate into society.^41^

## Data Availability

The data that support the findings of this study are available from the corresponding author, Z. B. Dombi, upon reasonable request.
